# Effectiveness of Test-Enhanced Learning (TEL) in lectures for undergraduate medical students

**DOI:** 10.12669/pjms.336.13358

**Published:** 2017

**Authors:** Aisha Ayyub, Usman Mahboob

**Affiliations:** 1 Dr. Aisha Ayyub, MBBS, PG Dip, M Phil (Pathology), CHPE, MHPE, Assistant Professor, Department of Pathology, KMU-Institute of Medical Sciences (KIMS), Kohat, Pakistan; 2 Dr. Usman Mahboob, MBBS, MPH, FHEA, DHPE, Assistant Professor in Medical Education, Institute of Health Professions Education & Research (IHPER) Khyber Medical University (KMU), Peshawar, Pakistan

**Keywords:** Learning, Medical education, Test-enhanced learning, Testing effect, Undergraduate lectures, Medical students

## Abstract

**Objective:**

To determine the effectiveness of Test-Enhanced learning as a learning tool in lectures for undergraduate medical students

**Method:**

This quantitative, randomized controlled trial included eighty-four students of 4^th^ year MBBS from Yusra Medical & Dental College, Islamabad. The duration of study was from March 2016 to August 2016. After obtaining the informed consent; participants were equally assigned to interventional and non-interventional study groups through stratified randomization. Single best answer MCQs of special pathology were used as data collection instrument after validation. A pre- and post-test was taken from both groups, before and after the intervention, respectively and their results were compared using SPSS version 21.

**Results:**

There were 13 male (31%) and 29 female (69%) participants in each study group who showed an equivalent baseline performance on pre-test (p=0.95). Statistically significant difference was found among mean scores of interventional and non-interventional study groups at exit exam (p=0.00). Interventional group also showed a significant improvement in their post-test scores (mean: 17.17±1.59) as compared to pre-test scores (mean: 6.19±1.81).

**Conclusions:**

Test-enhanced learning has significant effect on improving the learning of course content delivered to undergraduate medical students through lectures.

## INTRODUCTION

With continuous advancements in the medical education, various other instructional methodologies such as Small Group Discussions (SGD), Problem-based learning (PBL), Simulation-based learning and web-based learning, have been adopted to promote active learning among the undergraduate medical students.^1^ Although these strategies gained more attention because of motivating the active learning among students, the lectures still hold a central position for the transfer of knowledge as it combines content delivery and student interaction simultaneously.^2^ Especially at the undergraduate level, lectures are considered most economical, feasible and an equally effective method of imparting knowledge to a large group of students.^3^ However few researchers believe that lecture is a less effective teaching tool and provides passive environment for learning.^4^,^5^

Fortunately, literature from cognitive psychology offers some conceptual frameworks of learning theories which can help the medical educators to design such strategies that could promote better retention of information and thus facilitate the learning of taught content.^6^ Testing effect is one of those, which states that when the students are tested for a specific content, they remember it better and for longer time period than the content not tested at all.^7^ Tests not only strengthen the memory by retrieving the information but also improve the learning of contents through long-term retention of specific information. This phenomenon is known as *Test-Enhanced Learning*.^8^

Taking test is vexatious for both students and teachers; therefore the potential benefit of testing to facilitate the learning has been greatly overlooked in Pakistani educational setup and tests are merely used to evaluate the learning of undergraduate medical students in order to grade them in the class.^9^

Despite the promising advantages of Test-enhanced Learning (TEL) in cognitive psychology, its effectiveness as a learning tool in undergraduate teaching situations such as lectures has not yet been studied. Therefore, this study was designed to determine the effectiveness of Test-Enhanced Learning (TEL) as a learning tool in lectures for undergraduate medical students. The study findings will help the medical teachers to select TEL for their lectures at undergraduate level.

## METHODS

This quantitative randomized Controlled Trial (RCT) was carried out at Yusra Medical & Dental College (YM&DC), Islamabad, from March 2016 to August 2016, after obtaining ethical approval from the Ethical Committee of Khyber Medical University, Peshawar and Ethical Review Committee of YM&DC.

**Fig.1:**
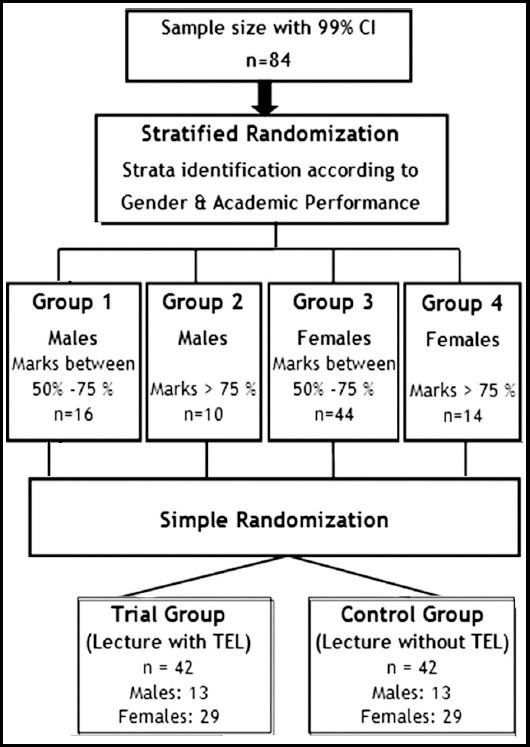
Randomization Technique used in the study.

The sample was calculated through OpenEpi sample size calculator^10^ at 99% confidence interval for exploratory, single centre interventional study. Eighty-four (n=84) medical students from Year-4 who were attending special Pathology lectures, were included in this study. Students absent from any lecture of special pathology or students getting 100% marks in pre-test were excluded out because their improvement in post-test could not be measured. After obtaining written informed consent from participants, stratified randomization was done according to gender and academic record (Fig.1).

A test comprising of sixty (60) single best answer MCQs on endocrinology module of special pathology was used as data collection instrument after validation. Both groups were given a same pre-test based on 20 single-best answer MCQs and each MCQ carried one (01) mark. The result of pre-test was recorded as base line score of each student.

After taking the pre-test, both groups were taught with respective methodology in split classes, having one topic of endocrinology module/week. The lecture content for both interventional and non-interventional group was identical and to avoid any lecture delivery bias, all lectures were delivered by a single teacher using Gagne events of instruction^11^ for standardization. Interventional group was taught through TEL and was exposed to five single-best MCQs after each lecture which were not included in pre- or post test, while control group was taught through lectures only.

At the end of endocrine module (4 weeks), both groups took a post-test consisting of 20 single-best answer MCQs with a gap of one week. These post-test MCQs were different from the pre-test MCQs but had the same difficulty level and carrying one mark each. (Total Marks = 20 each for pre-test and post-test).

After taking the post-test, in order to compensate for the expected TEL benefits, student of traditional teaching group were taught through TEL in special classes. Results of both tests were analyzed through SPSS-21. Between-subject difference in mean post-test scores of both groups was measured as primary outcome of the study (*p*<0.01).

## RESULTS

### Demographic characteristics of participants

Eighty four (n=84) undergraduate medical students, equally distributed in two groups (TEL &Lecture only group) through stratified randomization participated in this RCT. Each study group consisted of 13 male (31%) and 29 female (69%) participants. No statistically significant difference was found among mean ages of TEL group (23.02 ± 0.60) and Lecture only group (23.07±0.55) participants (*p* = 0.70).

### Between-subject difference in pre- and post-test scores

**Fig.2:**
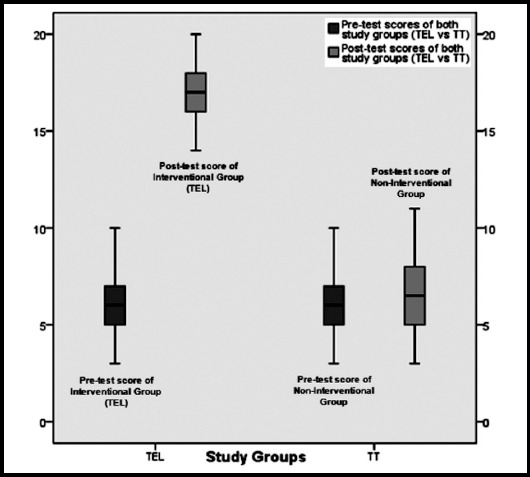
Between-subject difference in pre-test and post-test scores of both study groups.

Between-subject score difference among interventional (TEL) and non-interventional group was insignificant (*p* = 0.95) at pre-test and no group lagged behind the other. However, the main outcome of this study was between-subject difference in mean scores of post-test. Both groups showed statistically significant difference among their mean post-test scores (interventional group mean: 17.17±1.59 versus non-interventional teaching group mean: 6.52±2.02, *p* = 0.00). These results revealed that participants who were exposed to TEL intervention performed better than the participants of non-interventional group in their post-test (Fig.2).

### Academic performance of individual study group

**Fig.3:**
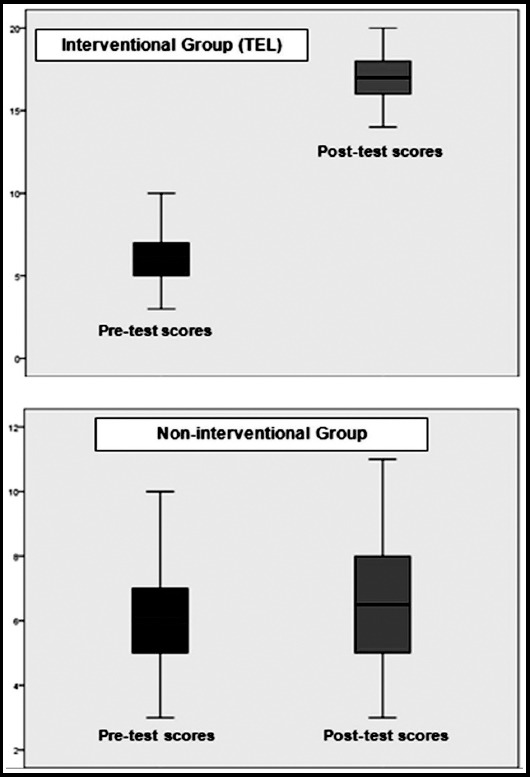
Comparison of within-subject difference of pre-test and post-test scores of interventional group (TEL) and non-interventional group.

A with-in subject difference of academic performance was also measured for each individual group using paired sample *t-*test as secondary outcome of this study. Participants of interventional group (TEL) demonstrated a statistically significant improved performance (*p* = 0.00) on post-test taken after the TEL intervention (Fig.3).

Study participants who were taught through lectures only, failed to demonstrate any statistically significant improvement in their post-test scores as compared to pre-test scores (*p* =0.057) (Fig.3).

Performance of male and female participants of interventional group was also compared. It was an interesting finding that both genders improved their post-test scores equivalently (mean post-test score of male students 17.92 ±1.25 versus mean post-test score of female students 16.83 ± 1.62, *p* = 0.03).

## DISCUSSION

Previous studies have observed the effect of TEL in different learning situations like e-seminars,^12^ CME conference^13^ and resuscitation skill course^14^ but not in lectures. Therefore, this study was aimed at assessing the effect of TEL on learning of content taught through lectures.

The results of our study demonstrated that incorporating TEL in pathology lectures enormously improved the learning of studied content among undergraduate medical students who were able to relate the underlying complex mechanisms with their endocrine disorders. This sound theoretical framework of basic science knowledge is also a pre-requisite to achieve excellence in future clinical reasoning and diagnostic skills.^12^,^15^

The results of our study are also supported by Rauph et al.,^12^ who demonstrated the positive effect of repeated testing in enhancing the long term retention of cognitive clinical concepts among undergraduate medical students. They found TEL a feasible and time-efficient methodology for teaching clinical medicine in seminars which substantially enhanced students’ learning outcomes.^12^

The TEL has also been found useful in undergraduate nursing education to promote learning and improved final performance than re-study of the same course contents.^16^ Findings of TEL effects in undergraduate pharmacy also support our results as they found improved performance of students on both, formative and summative assessment attributable to TEL methodology.^17^,^18^

Our study findings are also in accordance with the findings of study carried out by Baghdadi M,^15^ administered basic sciences MCQs tests as TEL methodology which in turn improved the learning of basic pathophysiology mechanisms among undergraduate dental students.^15^

In the present study, we compared the mean post-test scores of both interventional and non-interventional groups as the main outcome. Taking test as a learning exercise after each lecture increased the interventional group score remarkably as compared to non-interventional group score. Similar results in a previous randomized controlled trial, documented improved mean score of residents learning the didactic conference content through repeated testing versus the residents using repeated study as learning strategy. The improved learning was found to be due to active engagement of residents in TEL activities.^19^

In contrast to our findings, no such between-subject difference was found in a study where only a single test was given for learning activity of practicing physicians.^20^ In comparison, our study used four tests as learning exercise in lectures. Previous studies have established the relation between number of tests taken and improvement in long-term retention of knowledge.^13^,^19^,^21^,^22^ Thus, this discrepancy among results affirmed that single test is insufficient to retain knowledge for long-terms.

Our study also examined whether the improved performance of interventional group at post-test could be a gender-related phenomenon. The resultant findings did not establish such relationship as no significant difference was found among mean post-test scores of male and female participants. Inconsistent to our findings, a study,^23^ documented the male predominance in positive effects of testing and argued that these effects are related to increased cortisol level released in response to test anxiety in men.^23^ A possible explanation of these inconsistent findings is the context of testing as we introduced tests as a learning exercise and not for an assessment purpose.

### Strengths of the study

To the best of our knowledge, this study is first of its kind in Pakistan assessing the effectiveness of Test-enhanced learning in lectures for undergraduate medical students. The study design (RCT) with potentially eradicated selection and performance biases can be considered a robust one. We followed the current recommendations for this study.^19^,^24^ Equally spaced, repeated tests using multiple choice questions of higher cognitive level were taken over a six weeks span.

Unlike the previous medical education studies that evaluated the effectiveness of TEL in an artificial or simulated educational setting which can affect the actual learning process, the present study used real learning situation that is undergraduate pathology lectures. Other strength of the study was absence of any attrition that has affected the measure of TEL effectiveness in previous studies.^13^,^16^,^20^-^22^

### Limitations of the study

Despite being demonstrated the beneficial effects of TEL in undergraduate lectures, the generalisability of this finding is limited as study was conducted in only pathology discipline of one medical college. Further studies are needed to examine the effects of TEL in other disciplines.

We also could not follow the students’ performance in annual examination. For future studies, it will be beneficial if students can be followed up for a longer period of time so that a strong causal relationship can be established between repeated testing of taught content and long-term retention of that knowledge delivered through lectures.

## CONCLUSION

Test-enhanced learning has significantly improved the learning of course content delivered to undergraduate medical students through lectures. TEL can be used as an effective tool for promoting learning and enhancing the long term retention of taught content at undergraduate level. This would change the dynamics of interactive lecture without losing the focus of lecture’s content.

### Authors` Contribution

AA conceived, designed and did data collection, statistical analysis and manuscript writing.

UM did editing, review and final approval of manuscript.

## References

[1] Ilic D, Maloney S (2014). Methods of teaching medical trainees evidence-based medicine:a systematic review. Med Educ.

[2] Christopher DA (2011). Interactive large classes:The dynamics of teacher/student interaction. J Bus Econ Res.

[3] Papanna KM, Kulkarni V, Tanvi D, Lakshmi V, Kriti L, Unnikrishnan B (2013). Perceptions and preferences of medical students regarding teaching methods in a Medical College, Mangalore India. Afr Health Sci.

[4] Prober CG, Heath C (2012). Lecture halls without lectures—a proposal for medical education. N Engl J Med.

[5] Richardson D (2008). Don't dump the didactic lecture;fix it. Adv Physiol Educ.

[6] Roediger HL, Pyc MA (2012). Inexpensive techniques to improve education:Applying cognitive psychology to enhance educational practice. J Appl Res Mem Cogn.

[7] Butler AC, Roediger HL (2007). Testing improves long-term retention in a simulated classroom setting. Eur J Cogn Psychol.

[8] Roediger HL, Karpicke JD (2006). Test-enhanced learning taking memory tests improves long-term retention. Psychol Sci.

[9] Rauf A, Shamim MS, Aly SM, Chundrigar T, Alam SN (2014). Formative assessment in undergraduate medical education:concept, implementation and hurdles. J Pak Med Assoc.

[10] Dean AG SK, Soe MM OpenEpi:Open Source Epidemiologic Statistics for Public Health, Version.

[11] Hricko M (2008). Gagne's Nine Events of Instruction. Encyclopedia of Information Technology Curriculum Integration:IGI Global.

[12] Raupach T, Andresen JC, Meyer K, Strobel L, Koziolek M, Jung W (2016). Test-enhanced learning of clinical reasoning:a crossover randomised trial. Med Educ.

[13] Larsen DP, Butler AC, Aung WY, Corboy JR, Friedman DI, Sperling MR (2015). The effects of test-enhanced learning on long-term retention in AAN annual meeting courses. Neurology.

[14] Kromann CB, Jensen ML, Ringsted C (2009). The effect of testing on skills learning. Med Educ.

[15] Baghdady M, Carnahan H, Lam EW, Woods NN (2014). Test-enhanced learning and its effect on comprehension and diagnostic accuracy. Med Educ.

[16] Messineo L, Gentile M, Allegra M (2015). Test-enhanced learning:analysis of an experience with undergraduate nursing students. BMC Med Educ.

[17] Hernick M (2015). Test-Enhanced Learning in an Immunology and Infectious Disease Medicinal Chemistry/Pharmacology Course. Am J Pharm Educ.

[18] Horn S, Hernick M (2015). Improving student understanding of lipids concepts in a biochemistry course using test-enhanced learning. Chem Educ Res Pract.

[19] Larsen DP, Butler AC, Roediger HL (2009). Repeated testing improves long-term retention relative to repeated study:a randomised controlled trial. Med Educ.

[20] McConnell MM, Azzam K, Xenodemetropoulos T, Panju A (2015). Effectiveness of Test-Enhanced Learning in Continuing Health Sciences Education:A Randomized Controlled Trial. J Contin Educ Health Prof.

[21] Larsen DP, Butler AC, Lawson AL, Roediger HL (2013). The importance of seeing the patient:test-enhanced learning with standardized patients and written tests improves clinical application of knowledge. Adv Health Sci Educ Theory Pract.

[22] McConnell MM, St-Onge C, Young ME (2015). The benefits of testing for learning on later performance. Adv Health Sci Educ Theory Pract.

[23] Kromann CB, Jensen ML, Ringsted C (2011). Test-enhanced learning may be a gender-related phenomenon explained by changes in cortisol level. Med Educ.

[24] Roediger HL, Karpicke JD (2006). The power of testing memory:Basic research and implications for educational practice. Perspect Psychol Sci.

